# Polymer-dispersed liquid crystal elastomers

**DOI:** 10.1038/ncomms13140

**Published:** 2016-10-07

**Authors:** Andraž Rešetič, Jerneja Milavec, Blaž Zupančič, Valentina Domenici, Boštjan Zalar

**Affiliations:** 1 Department of Solid State Physics (F-5), J. Stefan Institute, Jamova 39, 1000 Ljubljana, Slovenia; 2 Jožef Stefan International Postgraduate School, Jamova 39, 1000 Ljubljana, Slovenia; 3 Dipartimento di Chimica e Chimica Industriale, Università degli studi di Pisa, via Moruzzi 13, 56124 Pisa, Italy

## Abstract

The need for mechanical manipulation during the curing of conventional liquid crystal elastomers diminishes their applicability in the field of shape-programmable soft materials and future applications in additive manufacturing. Here we report on polymer-dispersed liquid crystal elastomers, novel composite materials that eliminate this difficulty. Their thermal shape memory anisotropy is imprinted by curing in external magnetic field, providing for conventional moulding of macroscopically sized soft, thermomechanically active elastic objects of general shapes. The binary soft-soft composition of isotropic elastomer matrix, filled with freeze-fracture-fabricated, oriented liquid crystal elastomer microparticles as colloidal inclusions, allows for fine-tuning of thermal morphing behaviour. This is accomplished by adjusting the concentration, spatial distribution and orientation of microparticles or using blends of microparticles with different thermomechanical characteristics. We demonstrate that any Gaussian thermomechanical deformation mode (bend, cup, saddle, left and right twist) of a planar sample, as well as beat-like actuation, is attainable with bilayer microparticle configurations.

## Abstract (web summary)

Liquid crystal elastomers are promising for building actuators due to excellent thermomechanical response, but it is challenging to prepare them at macroscale with arbitrary shape. Here, the authors overcome this restriction by doping microparticles to the polymer matrix without employing mechanical stressing.

## Graphical abstract

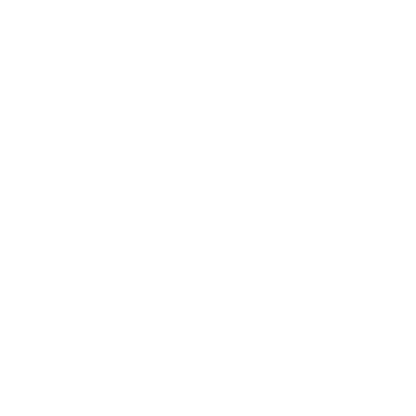

The widespread use of polymers and elastomers in modern technologies strongly relies on the fact that they are relatively undemanding for moulding, reshaping and machining. In most applications, these materials are used as passive mechanical components. Only recently, smart soft materials with external stimuli-induced mechanical responsiveness have been developed. Among them, electroactive polymers^1^, have been employed most extensively. Nevertheless, liquid crystal elastomers (LCEs) are becoming an increasingly strong competitor in the development of a new generation of actuator and transducer elements^2^, both for macroscopic devices, for example, artificial muscles, and micro- and nano-sized devices, for example, microelectromechanical systems (MEMS) and nanoelectromechanical systems (NEMS)^3^,^4^. In these smart polymer materials, changes in the molecular orientational order, equivalently nematic order, are manifested macroscopically as deformation of specimen geometry^5^. The control of nematic order and the consecutive morphing can be achieved by varying the temperature, either by contact heat conduction^6^,^7^, indirect heating via electromagnetic radiation-absorbing nanoparticle inclusions^8^,^9^,^10^, or by photoisomerization^11^.

Among the more exciting prototype devices^12^ exploiting shape memory behaviour of LCEs are microfluidic valves, Braille readers, holographic gratings and artificial cilia^13^,^14^,^15^,^16^. However, current LCE synthesis methods still lack easy implementation into larger scale production environment. The major obstacle is the microscopic size of LCE domains: although individual LCE domains possess shape memory, the sample as a whole is inert since conventional polymerization methods yield isotropic distribution of domain orientations. A crucial step of imprinting shape memory into a macroscopically-sized LCE specimen is, therefore, to instil permanent orientational alignment of domains, that is, an effectively ‘monodomain' state with anisotropic physical properties on the macroscopic scale. Currently, the most efficient way to achieve this is thermal polymerization of a mechanically stressed, partially crosslinked network^6^. Unfortunately, this procedure allows neither for preparation of samples of arbitrary shapes, nor for the miniaturization and templating of the geometry^17^,^18^. An alternative method that omits partial crosslinking has recently been invented^19^, but the alignment of domains still needs to be performed mechanically. Mouldable LCE materials for production of macroscopically sized monodomain parts of arbitrary shapes, that is, bulk parts with anisotropic thermomechanical properties, thus remain rather elusive. The availability of such materials could considerably expand the application scope of three-dimensional (3D) printing technologies^16^,^20^, which currently rely almost exclusively on isotropic raw materials. Moreover, shape-programmable^21^,^22^ 3D soft objects could be fabricated through imprinting spatially inhomogeneous thermomechanical or photomechanical anisotropy via curing in the presence of gradiented or voxelized (individually adjusted to small volume elements) non-mechanical external orienting field, using conventional fused deposition modelling and stereolithography additive technologies^20^,^23^. For two-dimensional (2D) elastic objects, the concept of programmable shape-changes has been demonstrated experimentally in thin elastic gel sheets with non-uniform shrinkage properties^24^ and in LCE films with voxelized nematic director field^25^, as well as explained theoretically in terms of non-Euclidean metrics-driven out-of-plane curving of planar sheets exposed to external stimuli^26^,^27^,^28^. Poor choice of suitable materials has so far impeded the extension of this concept to 3D.

In this paper, we present a new composite material, polymer-dispersed liquid crystal elastomer (PDLCE) that overcomes the above restrictions: it provides for moulding of soft elastomeric bulk or miniature, thermally morphable parts of any given shape. The main idea is to dope a conventional elastomer like polydimethylsiloxane (PDMS) with LCE microparticles (μLCEs) (Fig. 1a). Used as fillers or inclusions in a soft polymer matrix, μLCEs render the composite material effectively thermomechanically active, provided that their axes of anisotropy are at least partially aligned. The latter is accomplished by curing the mixture of polymer resin and μLCE in external magnetic field, exploiting the diamagnetic anisotropy of the μLCEs^29^. Since these are small with respect to the size of the composite specimen, inhomogeneities in local stress and strain can be disregarded, and the composite's effective elastic and thermomechanical properties resemble those of a conventional bulk, oriented LCE material. Unlike in competing approaches^6^,^19^, macroscopic thermomechanical anisotropy is imprinted into PDLCEs without mechanical stressing, using external orienting magnetic field, which imposes no restrictions on the shape and size of the specimen. In contrast to currently prevailing efforts of designing LCE composites where LCE is used as the matrix, functionalized with micro- or nanoparticles^8^,^10^,^30^, in PDLCEs it is μLCEs themselves that have the role of the colloid. We demonstrate that this paves the path towards designing thermomechanically functionalized conventional elastomers with tailorable shape memory and thermal expansion behaviour.

## Results

### Thermomechanical functionalizing with oriented μLCEs

**Figure 1:**
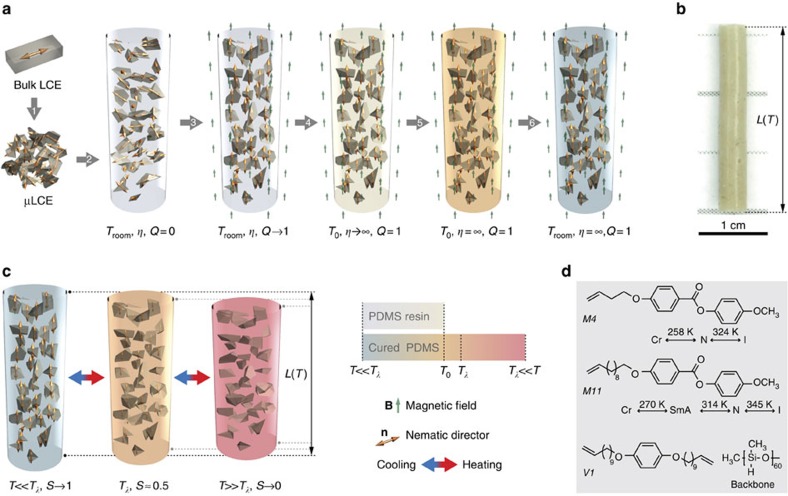
Preparation of a PDLCE composite. (**a**) Schematic illustration of the six-step PDLCE manufacturing method: (1) crushing the starting bulk LCE into μLCE particles, (2) dispersing the particles in uncured (low viscosity *η*) PDMS elastomer, (3) aligning the initially disordered μLCE in external magnetic field, that is, increasing μLCE orientational order from *Q*=0 to *Q*=1, (4) heating to setting temperature *T*_0_, (5) thermal curing of PDMS matrix at *T*_0_, and (6) cooling the resulting PDLCE composite to *T*_room_. (**b**) Photograph of a representative, cylindrically shaped *PDLCE-A* specimen, prepared according to the method shown in **a** (see Methods for composition and fabrication details). (**c**) Spontaneous mechanical deformation of PDLCE composite on crossing the nominal thermomechanical anomaly temperature *T*_*λ*_ of μLCE filler, associated with the change in the nematic order parameter *S*. Also shown is the colour bar used to colour-code the temperature of PDMS matrix in panels **a** and **c**. (**d**) Chemical structure of LCE material constituents: nematic *M4* and smectic A *M11* mesogenic side-chains with their respective bulk phase transition temperatures between the isotropic (I), nematic (N), smectic A (SmA) and crystalline solid (Cr) states, bi-functional crosslinker *V1*, and (poly)methylsiloxane backbone. For animated version of the μLCE alignment procedure (**a**) and of thermomechanical actuation (**c**) play the Supplementary Movie 1.

The availability of nematic monodomain μLCEs is mandatory for the design of PDLCEs. In view of the target application domain of PDLCEs, that is, moulding of macroscopically sized, thermomechanically functionalized soft objects that require abundant quantities of microparticles, recent breakthroughs in the synthesis of anisotropic colloidal μLCEs using templating^18^ or microfluidics^31^,^32^ approach offer a straightforward choice of particle production, but are technologically rather demanding as far as rapid, high-volume production is concerned. We propose a much simpler approach of low-temperature milling to freeze-fracture bulk LCE samples into micropowder with particle sizes in the 1–150 μm range (step 1 of Fig. 1a). It is assumed that μLCEs retain the thermomechanical behaviour of their bulk LCE parent, that is, *λ*_μLCE_(*T*)=*λ*_LCE_(*T*) ≡ *L*_LCE_(*T*)/*L*_LCE_(*T*_ref_), with the strain (*λ*_LCE_(*T*)−1)∝*S*(*T*) reflecting temperature dependence of the nematic order parameter *S*(*T*) associated with spontaneous orientational ordering of the mesogenic LCE network components^5^. In contrast to pure bulk liquid crystals, which typically exhibit sharp, weakly first order type *S*(*T*) anomaly at the clearing temperature *T*_NI_, in LCEs, where the mesogens are embedded into the network, the *S*(*T*) anomaly and hence the *λ*_LCE_(*T*) anomaly is smeared about the nominal thermomechanical transition temperature *T*_*λ*_ (ref. 33). The degree of smearing and the shift of *T*_*λ*_ with respect to *T*_NI_ depend on structural parameters like, for example, concentration of crosslinkers^34^. The reference specimen length *L*_LCE_(*T*_ref_) is measured in the isotropic phase at *T*_ref_≫*T*_*λ*_ where *λ*_LCE_(*S*→0)→1.

Individual PDLCE specimens discussed here are labelled sequentially as *PDLCE-β*. The hyphenated sequential suffix *β*=*A*, *Ap*, *B*, *B1*/*B2*, *C*, or *Cp* is associated with the type of embedded μLCE particles. These are labelled as *μLCE-α*, with *α*=*A*, *Ap*, *B1*, *B2*, *C*, or *Cp* denoting chemical composition and fabrication parameters of their respective bulk *LCE-α* parents (see Methods for details on the fabrication procedure and Table 1 for parameters associated with the *α* suffix). All investigated PDLCEs contain a single type of μLCE particles, equivalently *β*=*α*, except for *PDLCE-B* composed with a mix of *μLCE-B1* and *μLCE-B2*, and *PDLCE-B1/B2* composed of fused *μLCE-B1* and *μLCE-B2* layers. Secondary index *p* in *Ap* and *Cp* is used to mark polydomain bulk LCE material.

Although monodomain bulk LCE pieces (*LCE-A*, *LCE-B1*, *LCE-B2*, *LCE-C*) represent the optimal choice that guarantees individual grains of the powderized material to retain nematic monodomain state (Supplementary Fig. 1 and Supplementary Note 1), polydomain bulk LCE starting material (*LCE-Ap*, *LCE-Cp*) can be used as well, since the specimens are crushed into microparticles of the size of nematic domains, typically several μm (ref. 5). Subsequently, microparticles are dispersed into conventional liquid elastomer resin, favourably in a 1:1 particle/matrix weight ratio (step 2 of Fig. 1a). A specific selection of thermally curable PDMS as the matrix material provides for chemically inert, appropriately viscous environment for efficient mixing and negligible particle aggregation on the timescale of the curing process.

**Figure 2:**
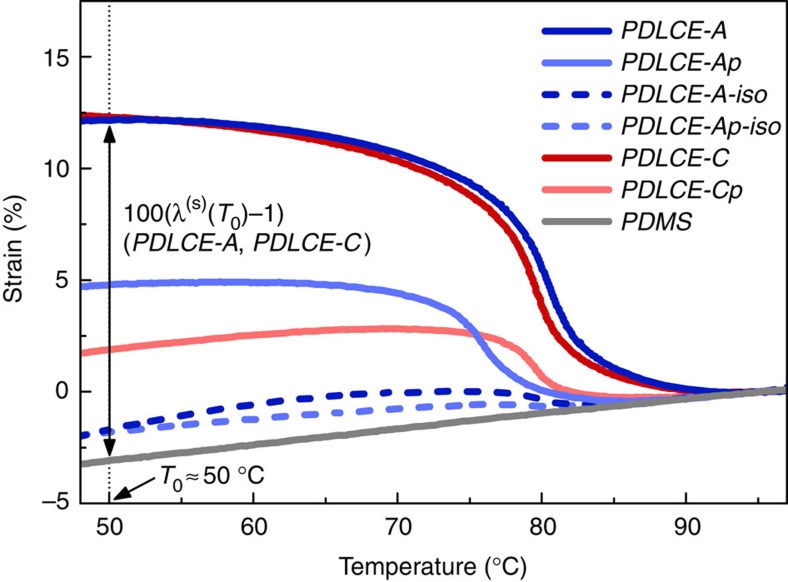
PDLCE thermomechanical response curves. Experimental *λ*(*T*)=*L*(*T*)/*L*(*T*_ref_) profiles for PDLCE composites listed in Table 2 are shown as percent strain 100(*λ*−1). The reference temperature *T*_ref_ is 95 °C. Specimens with *Q*>0 (solid lines) clearly display expansion on decreasing temperature, with strain anomalies at *T*_*λ*_≈80 °C reminiscent of pure monodomain LCEs. Low-temperature strains *λ*(*T*_0_) span from at least several per cent for partially aligned samples (*Q*<1) to about 12.5%, equivalently *λ*=1.125, for the best-performing *PDLCE-A* and *PDLCE-C* in which μLCE particles are fully aligned (*Q*→1). On the other hand, isotropic PDLCEs (*Q*=0, dashed lines, suffix ‘*-iso*') only display a small residual anomaly at *T*_*λ*_ and contraction on decreasing temperature, with *λ*(*T*_0_)≈0.98. Compensated for the conventional linear thermal expansion with coefficient *α*_PDMS_ (PDMS curve, grey solid line, with *λ*_PDMS_(*T*_0_)≈0.97), the partial thermomechanical response *λ*^(*S*)^(*T*)=*λ*(*T*)−*α*_PDMS_(*T*−*T*_ref_) (double arrow line at *T*_0_ for *PDLCE-A* and *PDLCE-C*), arising from LCE microparticles' nematic order *S* (see the modelling in Supplementary Note 3), amounts at *T*_0_ to a fair *λ*^(*S*)^≈1.16 for *PDLCE-A* and *PDLCE-C*, whereas it vanishes (*λ*^(*S*)^≈1.01), as anticipated, for isotropic PDLCEs.

Macroscopic shape memory is rendered into the composite material by orienting the prepolymer dispersion of μLCEs in the external magnetic field of magnitude *B*=|**B**| and subsequently locking the orientations by thermal curing of the matrix at elevated temperature *T*_0_ (steps 3 to 5, respectively, of Fig. 1a). The degree of diamagnetic anisotropy-driven μLCE alignment^35^ is quantified by the orientational order parameter 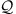 (ref. 36), which is rather independent of temperature since μLCE orientational distribution is locked during the setting of the resin. 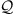 is to be distinguished from the nematic order parameter *S*(*T*) measuring molecular orientational order within a nematic domain and exhibiting strong temperature dependence (see above). At moderate magnetic fields (*B*≈1 T), saturation of 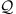 occurs within several minutes, whereas at higher, nevertheless readily available fields of several T, it only takes a few seconds with our particular choice of materials^37^ (for details on magnetic alignment dynamics see Supplementary Fig. 2 and Supplementary Note 2). Moreover, the aligning efficiency depends very little on the shape of microparticles^38^. Such a rapid magnetic manipulation of bulk quantity PDLCE material thus offers a competitive advantage over existing techniques that rely on mechanical manipulation. The effective thermomechanical response *λ*(*T*)=*L*(*T*)/*L*(*T*_ref_) of the resulting cured PDLCE, depicted schematically in Fig. 1c, is shown in Fig. 2 for a selection of specimens. *λ*(*T*_0_) performance figures of all investigated PDLCEs are given in Table 2. The observed response, typically *λ*(*T*_room_)≈*λ*(*T*_0_)≈1.12 for *PDLCE-A* and *PDLCE-C*, is reduced with respect to the response of bulk monodomain LCE where *λ*_LCE_(*T*_room_)≈1.45, due to the presence of thermomechanically inactive PDMS matrix (see the modelling of effective response in Supplementary Note 3).

Structurally, the obtained composites are analogous to polymer-dispersed liquid crystals where the nematic director of liquid-crystalline microdroplets, embedded in a polymer matrix, is manipulated by external fields or temperature to control the optical anisotropy of the system^39^. In PDLCEs presented here, μLCEs, embedded in an elastomer matrix, can be manipulated in a similar fashion to control the elastic anisotropy and mechanical dimensions of the sample. These can be fine-tuned by using μLCEs composed of different monomer species^40^, like for example, *M4* and *M11* in the present study (Fig. 1d).

**Figure 3:**
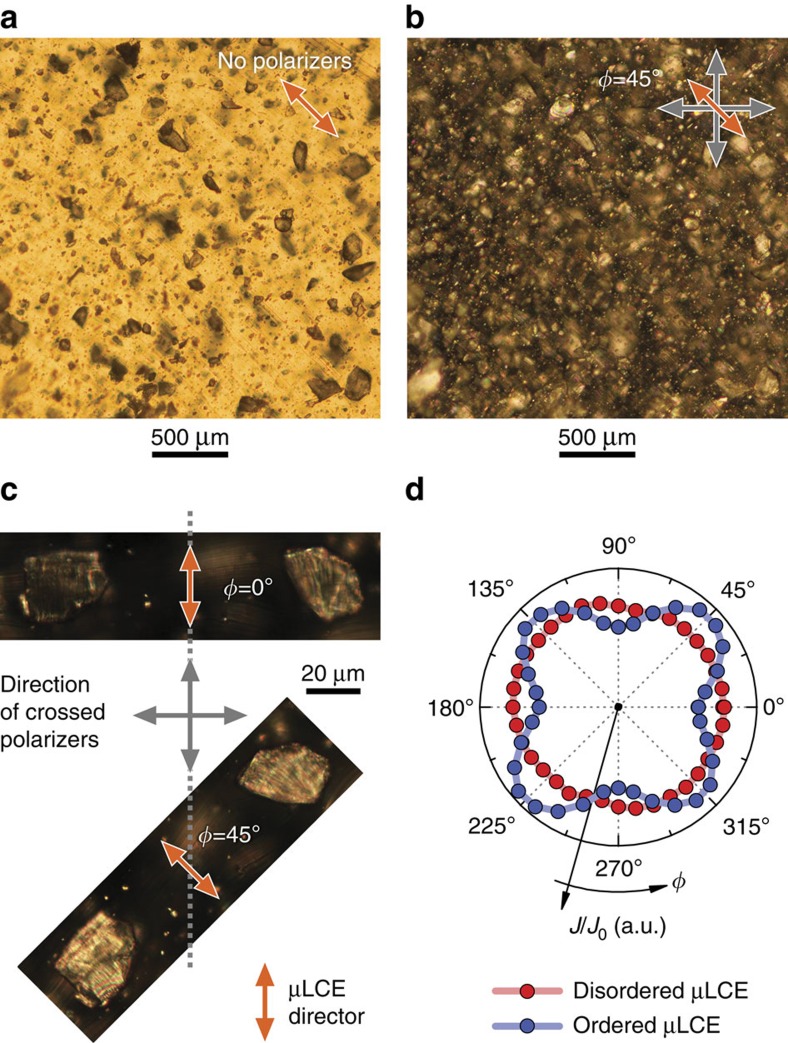
Structure of PDLCE as seen under polarizing optical microscope. (**a**) Without polarizers, the isotropic matrix transmits light, whereas LCE microparticles are seen as dark spots. (**b**) Under crossed polarizers, the light passing through the matrix is blocked, whereas LCE microparticles do transmit light due to their anisotropic nature. (**c**) Two microparticles compared at different orientations with respect to polarizers. The particles are dark when their directors are aligned with one of the polarizers (*ϕ*=0°) and bright at *ϕ*=45°. *PDLCE-A* sample, cut into 0.1 mm thick slice, with low, 5% wt concentration of *μLCE-A*, was used for microscopy investigations. (**d**) Polar plot of the sample's transmittance *J*/*J*_0_ demonstrating the optical anisotropy of the aligned (ordered) sample, arising from the alignment of μLCE particles.

The main characteristics of PDLCE structure can easily be observed under polarizing optical microscope. Depending whether crossed polarizers are used or not, the particles are seen as dark (Fig. 3a) or bright spots (Fig. 3b), respectively. In spite of a relatively wide distribution of particles' size, their dispersion inside the matrix is rather homogeneous. The alignment of particles was verified by reorienting the sample with respect to the polarizers. Specifically, the magnified view of Fig. 3c reveals synchronous changes in the brightnesses of an arbitrary pair of particles on changing the orientation angle *ϕ*, indicating that nematic directors are well aligned on the microscopic scale. Macroscopic alignment uniformity of field-aligned μLCE is demonstrated in Fig. 3d via anisotropic (*ϕ*−dependent) overall light transmittivity; the latter is isotropic in PDLCE with disordered μLCE.

### Shape-change programming of PDLCEs

**Figure 4:**
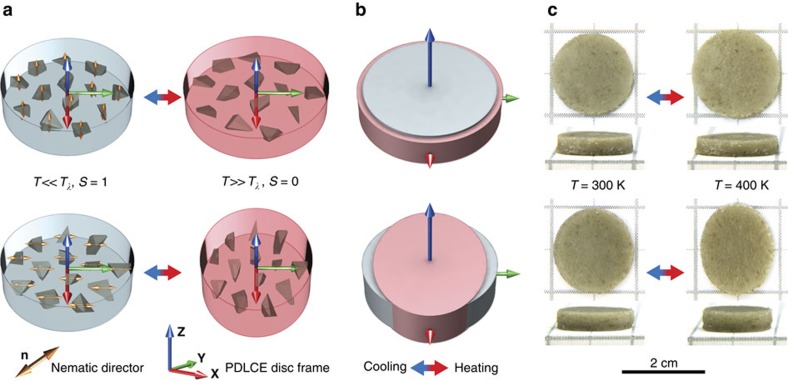
Morphing behaviour of PDLCEs with uniform LCE microparticle alignment. (**a**) Illustration of thermal actuation of PDLCE disks, their axes of symmetry denoted by **Z**, with nematic directors **n** oriented ‘out-of-plane' (top) and ‘in-plane' (bottom), that is, with **n**||**Z** and **n**⊥**Z**. On heating to *T*>*T*_*λ*_ (blue-to-red arrow, also for **c**), the LCE material becomes isotropic (change of the nematic order parameter *S* from 1 (large orange double arrow) to 0 (vanishing orange double arrow) accompanied by contraction of μLCE particles along the nematic director **n**). The temperature of PDMS matrix is colour-coded in accordance with the colour bar of Fig. 1 (blue-cold, red-hot). Note that *S*=1 corresponds to ideal nematic order, never found in a real system. Deuteron NMR, performed on *LCE-A* with benzene-ring-labelled *M4,* yields *S*≈0.65 at *T*_room_ (ref. 51). (**b**) Macroscopically observed effective thermomechanical response of PDLCE composite: oblate deformation of the **n**||**Z** disk (top) and prolate deformation of the **n**⊥**Z** disk (bottom). (**c**) Top- and side-view photographs of **n**||**Z** (top) and **n**⊥**Z** (bottom) *PDLCE-A* disks at *T*=300 K<*T*_*λ*_ and *T*=400 K>*T*_*λ*_, proving the concept of programmable PDLCE shape memory.

Probably the most applicable feature of PDLCEs is the ability to program their shape memory behaviour, beyond the currently prevalent contraction/dilatation and bending-style reshaping^2^. In the simplest case, this can be achieved by controlling the orientation of μLCE during the curing phase. In order to demonstrate this, we manufactured two identically shaped *PDLCE-A* disks with different orientation of nematic director **n** with respect to disk symmetry axis **Z**, one with an ‘out-of-plane' and the other with an ‘in-plane' orientation (Fig. 4a). On actuating the particles by raising *T* above *T*_*λ*_, an isovolumetric shape change thins the **n**||**Z** disk and increases its diameter, whereas it thickens the **n**⊥**Z** disk and makes it elliptically shaped (Fig. 4b,c).

**Figure 5:**
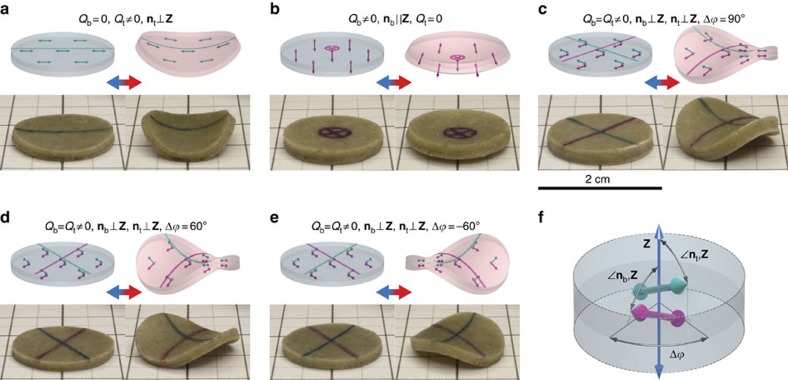
Programmable shape memory of PDLCEs with bilayer director field. Desirable thermomechanical response is instilled by configuring μLCE orientational order *Q* and orientation of nematic director **n** in the top and bottom PDLCE layers (indices t and b), respectively. All basic thermomechanical actuation modes, specifically bend deformation (**a**), cup deformation (**b**), saddle deformation (**c**), as well as left twist (**d**) and right twist (**e**) deformation can be realized by a suitable choice of *Q*_t_ and *Q*_b_, of the orientation of **n**_t_ (cyan double arrows) and **n**_b_ (magenta double arrows) with respect to the surface normal **Z** of the sample, as well as of the relative azimuth Δ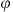 between **n**_t_ and **n**_b_(**f**). In each of panels **a**–**e**, the particular μLCE configuration and anticipated sample shape is depicted schematically in the two icons at the top, with the left one representing low-temperature state (*T*<*T*_*λ*_, blue-tinted PDMS matrix) and the right one representing high-temperature state (*T*>*T*_*λ*_, red-tinted PDMS matrix). Temperature-controlled shape morphing of real *PDLCE-A* bilayer samples is demonstrated with respective photographs at the bottom of panels **a**–**e**, the left one showing the specimen at *T*=300 K and the right one showing the specimen at *T*=400 K. Cyan (top layer) and magenta (bottom layer) solid lines and cross-hairs mark the respective directions of macroscopic thermomechanical anisotropy axes.

It is also straightforward to imagine morphable objects of higher complexity, produced in a similar way by spatially modulating the direction and/or the magnitude of **B**, for example, by additive layer manufacturing of partially polymerized layers^41^. In general, by controlling the spatial profile of the μLCE director field, the final, thermomechanically inhomogeneous sample could exhibit arbitrary deformations on temperature changes. We demonstrated this functionality by fabricating bilayer discs of various director configurations, resulting in, on external heat stimuli, curved shapes with all possible combinations of principal curvatures, that is, with positive and negative values of respective Gaussian curvatures^22^ at disc centres (Fig. 5, animated version in Supplementary Movie 2). The observed shapes are reminiscent of the ones recently predicted for thin nematic elastomer sheets with inhomogeneous director field^26^, realized by surface-aligning the voxels of inherently 2D specimen^25^. In our case, however, we address the programming of thermomechanical response of 3D objects since our fabrication approach imposes little restrictions on the shape and size of the sample and on the spatial configuration of nematic director. Moreover, our approach of creating such objects by fused deposition modelling of multilayer PDLCEs with compositionally identical matrix/μLCE layers may prove advantageous over fabricating hybrid structures with laminated substrate/LCE layers^42^, as well as over gel lithography where the programming of buckling behaviour is achieved by voxelizing swelling response^43^. Also notable is that in our simple scenario of a bilayer structure, any desired Gaussian curvature of the surface of the object can be established with a rather trivial nematic director configuration, in contrast to 2D case where relatively complex patterns of **n** (ref. 26) are required. We achieved this by programming distinct values of **n** and in each layer while keeping these values homogeneous within a layer (Fig. 5). Another intriguing feature, pertaining to 3D-character of **n**, is that the handedness of thermomechanically deformed object can be controlled by the handedness of chiral thermomechanical anisotropy field (Fig. 5d,e). Contrary to recent applications where this field is homogeneous across the sample and where morphing is controlled by spatially inhomogeneous external fields^44^, PDLCEs allow for homogeneous external stimuli since shape-change response is intrinsic to the specimen owing to spatially inhomogeneous thermomechanical anisotropy field.

### Designing bimodal shape-change behaviour

**Figure 6:**
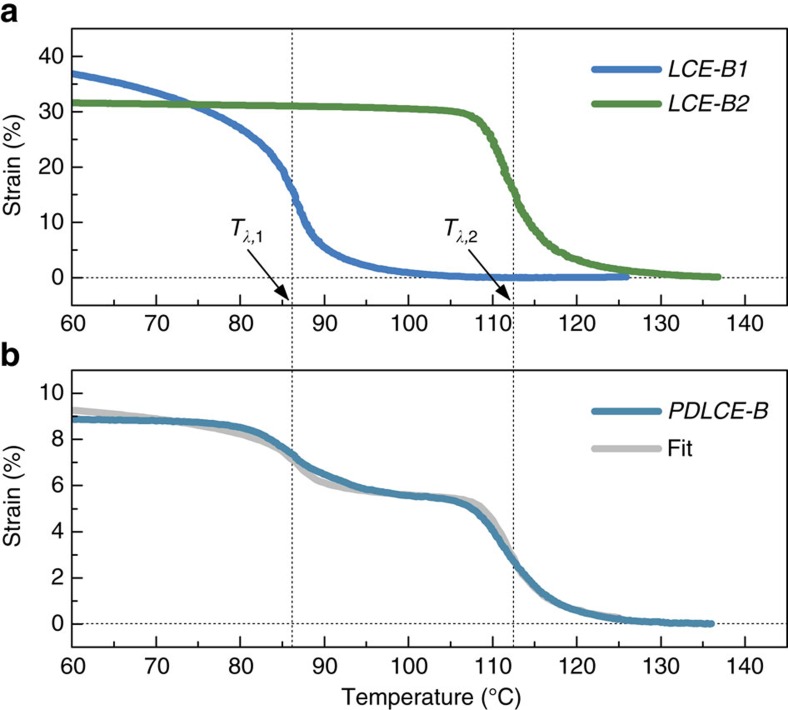
C**ustomization of thermomechanical response.** (**a**) Thermomechanical, equivalently strain versus *T* response curves of nematic *LCE-B1* and smectic A *LCE-B2* used for preparation of the bimodal PDLCE composite. (**b**) Experimentally determined bimodal thermomechanical response of *PDLCE-B*, fitted by linear superposition model. Percentage strain 100(*λ*(*T*)−1) is shown in (**a**,**b**).

Since PDLCEs are effectively binary soft-soft composites, they exhibit elastic behaviour intermediate between the rubber elasticity of the cured matrix and soft/semi soft elasticity^5^ of μLCE. Properties of the final composite can thus be tailored by varying the concentration of particles, particle size and the type of particles or matrix material. More than one LCE type can be used to prepare composites with advanced features. In multicomponent PDLCE composites, the elastic and thermomechanical properties are determined by the relative concentrations and the individual properties of the constituting species. Qualitatively, the effective response of the composite should behave as a superposition of responses of individual components. Specifically, in PDLCEs made of two-component μLCE blend, the overall thermomechanical response should amount to *λ*(*T*)=*k*_1_*λ*_1_(*T*)+*k*_2_*λ*_2_(*T*). *k*_1_ and *k*_2_ are the weights of respective individual LCE species. We have experimentally verified this assumption by preparing *PDLCE-B*, comprising of blended *μLCE-B1* and *μLCE-B2*, in 1:1 wt ratio. Their respective bulk LCE parents, *LCE-B1* and *LCE-B2,* exhibit well-separated temperatures of thermomechanical anomalies, with *T*_*λ*,2_−*T*_*λ*,1_≈25 °C (Fig. 6a). Individual *T*_*λ*_ can be fine-tuned by adjusting the ratio of the *M4* to the *M11* mesogen (Fig. 1d), with the limiting values *T*_*λ*,min_≈80 °C for 100% *M4*-based bulk LCE (*LCE-A*) and *T*_*λ*,max_≈120 °C for *M11*-only bulk LCE (not used in this study)^40^. The *λ*(*T*) profile of *PDLCE-B* clearly exhibits bimodal behaviour (Fig. 6b), with two anomalies arising from the two distinct phase transitions associated with the two microparticle species. The optimal fit is obtained with *k*_1_=0.1 and *k*_2_=0.18. We attribute the stronger impact of the *μLCE-B2* component (*k*_2_>*k*_1_) to its much higher Young's modulus (*E*_2_=3.2 MPa as compared with *E*_1_=230 kPa of the *μLCE-B1*). This is so, since, in addition to orientational ordering of mesogenic molecules (nematic phase) below *T*_*λ*,1_ in the *M4*-rich *LCE-B1*, molecular layers perpendicular to nematic director (smectic A phase) are formed below *T*_*λ*,2_ in the *M11*-rich *LCE-B2*, and these are less prone to mechanical deformation^5^.

**Figure 7:**
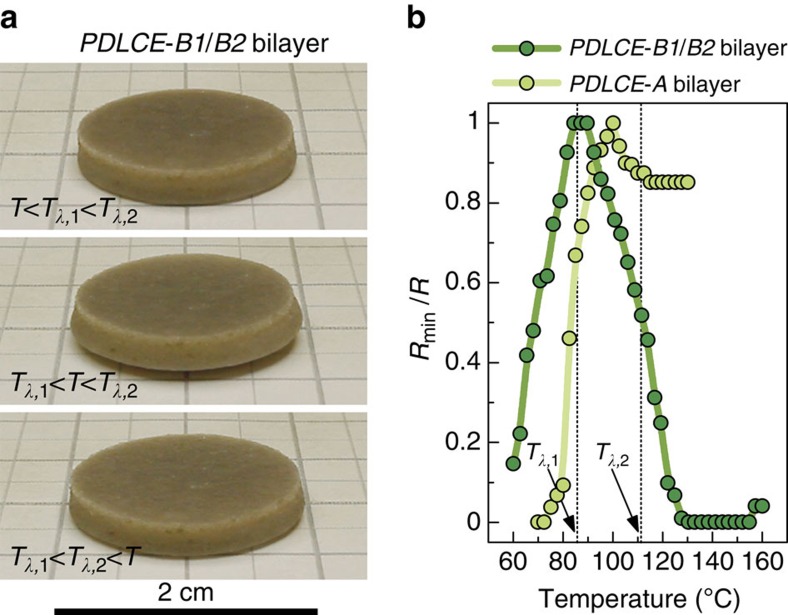
Beat-like thermomechanical actuation. (**a**) Photographs of cup-shape morphing behaviour in a bilayer PDLCE sample *PDLCE-B1/B2* in which the top layer is made of *PDLCE-B1* and the bottom layer of *PDLCE-B2*, both with **n**||**Z**. The difference in *T*_*λ*_ between the two layers leads to strong mismatching of local strains at the layer interface at temperatures intermediate between *T*_*λ*,1_ and *T*_*λ*,2_, compensated by non-planar deformation of the specimen (**a**, middle). For comparison, the **n**||**Z** monolayered *PDLCE-A* specimen of Fig. 4 remains planar in the whole temperature range. (**b**) Comparison of the step-like thermomechanical actuation of a bilayer *PDLCE-A* sample (for example, cup-shaped specimen of Fig. 5b) and of the beat-like thermomechanical actuation of bilayered *PDLCE-B1/B2*, using the relative surface curvature *R*_min_/*R* as the measure of mechanical deformation.

The potential of PDLCEs for programming non-monotonous thermomechanical response, more intricate than conventional smeared step-like one, is further demonstrated using bilayer sample geometry, discussed above. Specifically, the specimen *PDLCE-B1/B2* comprising of fused *PDLCE-B1* and *PDLCE-B2* layers is planar at high and low temperatures, but exhibits cup-shaped out-of-plane deformation in a beat-like fashion on low–high temperature stepping (Fig. 7 and Supplementary Movie 3).

### Controlling effective thermomechanical and elastic response

**Figure 8:**
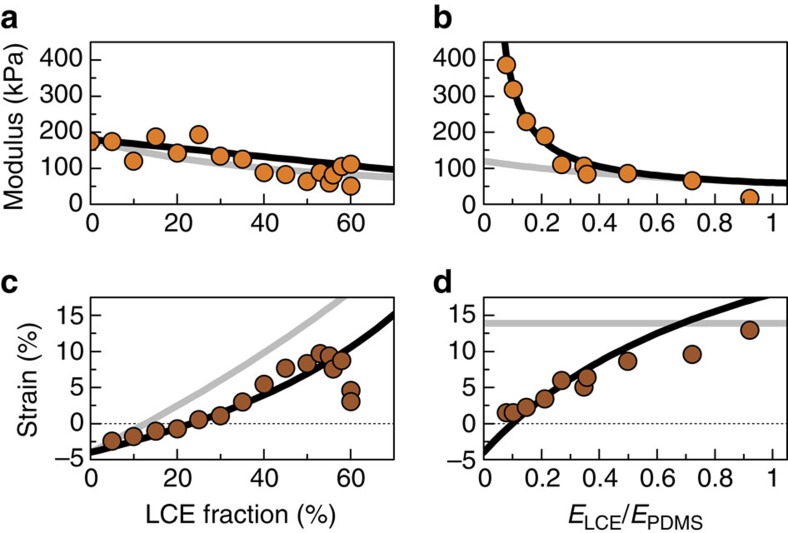
Optimization of properties of PDLCE composites. Experimental values of *E* (orange circles) and percentage strain 100(*λ*(*T*_room_)−1) (brown circles) were measured for *PDLCE-A* at *T*_room_ as functions of LCE material fraction *ν* (**a**,**c**) and its relative elastic modulus *y*=*E*_LCE_/*E*_PDMS_ (**b**,**d**). *μLCE-A* thermomechanical dopant exhibited *λ*_LCE_(*T*_room_)=1.45±0.05 strain and *E*_LCE_=60±6 kPa Young's modulus. *ν*-dependence points were obtained with composites prepared with matrix material of Young's modulus *E*_PDMS_=180±15 kPa, whereas *y*-dependence points were obtained by adjusting the PDMS matrix modulus *E*_PDMS_ via base/hardener composition at a constant *ν*=0.5, equivalently 50%, LCE fraction. Solid lines show the theoretical predictions from the ‘series' (light grey) and the ‘parallel' (black) model, with evidently favoured ‘parallel' scenario.

A simple quantitative analysis of PDLCE behaviour can be made by considering combined elasticity models (Supplementary Fig. 3 and Supplementary Note 3), providing for an estimate of the upper and lower limits of composite's effective Young's modulus *E* and thermomechanical response *λ* in terms of ‘series' scenario (alternating matrix and filler layers orthogonal to **n**) and ‘parallel' scenario (layers parallel to **n**)^45^. The ‘parallel' model predictions, calculated by solely considering the individual properties of the matrix and filler materials, not by fitting, are in particularly good agreement with the experimental results of the measurements of PDLCE's *E* and *λ* as a function of composition parameters, specifically the LCE material fraction *ν* and relative elastic modulus *y*=*E*_LCE_/*E*_PDMS_ (Fig. 8a–d). This can be efficiently utilized for tailoring elastic and thermomechanical properties of PDLCEs. The optimal region for maximizing the strain response of the composite is 40–60 wt% filler (*ν* within 0.4 and 0.6, that is, about 1:1 μLCEs versus PDMS ratio), as determined experimentally from the saturation of the strain in Fig. 8c. The deviation of experimental points from the prediction in the strain versus LCE fraction plot for *ν*>0.6 can be attributed to percolation of μLCEs, which prevents the alignment in the external field. We note that μLCEs, produced by freeze-fracturing, do not have controlled shapes and are not homogeneously sized, so that using smooth-surfaced^32^, optimally spherical^31^, particles might result in increased thermomechanical performance, with the theoretical limit of 18% maximal effective strain for *ν*=0.74 that corresponds to close-packed equal spheres.

The highest achieved strain at *T*_room_ is about 12% in the 50% LCE fraction *PDLCE-A*, at *E*≈100 kPa modulus. This may seem as a rather modest performance, particularly in view of targeting the performance of pure materials, that is, the 45% (*λ*_LCE_(*T*_room_)≈1.45) typical strain of *LCE-A* and *E*_PDMS_≈1 MPa typical modulus of PDMS. However, strains below 10% are quite adequate for many shape-programming applications^46^ and lead to substantial buckling, provided that material's internal strain matrix is properly programmed, as evidently demonstrated with bilayer PDLCEs (Fig. 5). Moreover, the proposed methodology for preparing PDLCEs imposes practically no restrictions on the choice of matrix and filler materials. Selection e.g., of a main-chain μLCE as a thermomechanical filler, with *λ*_LCE_(*T*_room_)≈2 and *E*_LCE_≈1 MPa (ref. 47), should result in an excellent thermomechanical and elastic performance, *λ*(*T*_room_)>1.5 and *E*>1 MPa, as inferred by relations for *E* and *λ*(*T*) for the ‘parallel' model.

### Comparing performance of PDLCEs to conventional LCEs

Strain versus temperature curves, presented in Fig. 2, reveal that thermomechanical response in the form of *λ*(*T*) anomaly, although in general somewhat suppressed with respect to *PDLCE-A* where monodomain LCE bulk material is used, is even observed in PDLCEs with magnetic field-aligned μLCEs made either of conventionally two-step crosslinked^6^ polydomain bulk LCE (for example, *PDLCE-Ap* containing *μLCE-Ap*), or of one-step crosslinked polydomain bulk LCE (for example, *PDLCE-Cp* containing *μLCE-Cp*). Obviously, the freeze-fracturing approach is sufficiently effective in generating μLCEs small enough to possess diamagnetic anisotropy and thus to become field-reorientable as a whole (see Supplementary Note 1). Even more notably, when μLCEs are fabricated from bulk LCE that is one-step crosslinked in high magnetic field (*LCE-C*, cured in 12 T magnet), the resulting composite material (*PDLCE-C*) performs thermomechanically on par with *PDLCE-A*. Evidently, *μLCE-C* particles are nematic monodomains and can be successfully used in place of *μLCE-A* particles, with the advantage of eliminating the second crosslinking step, that is, external mechanical straining, from the fabrication process of their bulk LCE parent. Availability of one-step crosslinked bulk parent materials like *LCE-C* further enhances the mass-production potential of μLCEs.

No attempt at quantifying the residual soft elasticity^5^ of PDLCEs has been made, but the opportunity of investigating this phenomenon should not be ignored, particularly in view of multitude of possible geometries, that is, easily accessible extension, compression and shear deformation modes. As also evident from Figs 1b, 4c, 5a–e and 7a, filling an intrinsically transparent PDMS with μLCE, made of transparent bulk monodomain LCE, results in an opaque PDLCE specimen. To diminish this light-scattering-inflicted disadvantage, μLCE material of either nanometric dimensions or a composite with refractive index of thermomechanically active microparticles matched with the index of the matrix should be used^48^. Although the potential of freeze-fracturing and microfluidics techniques for high-throughput production of nano-LCEs is yet to be investigated, we find these approaches more promising than index matching, since the latter imposes serious restrictions on the choice of matrix and filler materials. We conjecture that microsized and thin film PDLCE objects, composed of nano-LCEs as thermomechanical fillers and photodefinable matrix^49^, could readily be prepared by employing the already existing arsenal of polymer micromoulding technologies^50^.

## Discussion

Here we demonstrate that the common and prevailing, nevertheless rather tedious approach of inventing new chemistry, in order to marry, within a single material, thermomechanical responsiveness of LCEs with manufacturing advantages of conventional thermosetting or photosetting polymers, can be overcome with PDLCEs. These novel composite soft materials exploit an idea of thermomechanical doping, that is, the fact that a fair degree of thermomechanical activity of the embedded microparticles can be propagated into the effective response of the elastomer matrix, provided that microparticles are aligned. Our specific choice of PDMS matrix and μLCE dopant could be replaced with any choice of compatible soft materials, an inert isotropic elastomer as the matrix and thermomechanically or photomechanically responsive microparticles as fillers. Very importantly, functionalization, for example, with electrically conductive, thermally conductive, electromagnetic radiation-absorbing, ferroelectric and ferromagnetic particles can be performed on the matrix material rather than on the LCE material. The new concept of simple thermomechanical activation of conventional isotropic elastomers, introduced in this study by developing PDLCEs, opens up exciting new possibilities for future additive manufacturing technologies targeted towards 3D printing of thermally- or photomorphable artefacts.

## Methods

### Fabricating bulk LCE materials

Bulk LCE materials were polymerized by employing the standard two-step crosslinking approach^6^, consisting of partial crosslinking of prepolymer resin in thermally stabilized centrifuge and subsequent crosslinking of the sample exposed to mechanical stress, typically *σ*≈100 kPa (*LCE-A*, *LCE-B1*, *LCE-B*2), in the oven. *LCE-C* bulk material was one-step polymerized in the cryostat of a *B*=12 T superconducting magnet. Polydomain bulk material *LCE-Ap* was obtained by omitting external mechanical stress in the second crosslinking step. Similarly, the absence of external magnetic field gave rise to polydomain bulk *LCE-Cp*. Networks were crosslinked with 15 mole % of *V1* (percentage with respect to *M4*, *M11* and *V1* monomer total). *LCE-A* and *LCE-C* exhibiting nematic phase below *T*_*λ*_≈80 °C were prepared with nematogenic *M4*, whereas *LCE-B1* with nematic phase below *T*_*λ*,1_≈87 °C and *LCE-B2* with smectic A phase below *T*_*λ*,2_≈112 °C were prepared with *M4*_0.6_*M11*_0.4_ and *M4*_0.2_*M11*_0.8_ respective compositions of *M4* and smectogenic *M11* (ref. 40).

### Fabricating PDLCE composites

Bulk materials were first cut into smaller pieces (1 mm^3^) to prepare for freeze-milling in a mortar. The milling process was performed with a mix of LCE pieces and with liquid PDMS base (Sylgard 184 silicon elastomer kit) in 3:1 weight ratio. The LCE/PDMS mixture was frozen by repeatedly pouring liquid nitrogen over it. A pestle was used to crush the LCE/PDMS mixture into smaller size pieces for as long as the mixture remained frozen. This process was repeated until the emerging paste became homogeneous, containing small enough μLCEs. Water condensate was removed by drying at *T*_room_. Subsequently, PDMS resin of appropriate weight and base/hardener ratio was added in order to obtain the μLCE/PDMS prepolymer mixture of a desirable μLCE mass weight *ν* and cured PDMS Young's modulus *E*_PDMS_. After being evacuated to get rid of entrapped air, the final mix of uncured PDMS and μLCE, typically 80 mg in mass, was introduced, using a spatula, into a glass tube (50 mm length, 3 mm inner diameter). The inner surface of the tube was covered with a thin Teflon sleeve to prevent PDMS adhesion to the glass. The partially filled tube, sealed with Teflon tape on both sides, containing cylindrically-shaped, uncured and non-aligned PDLCE specimen, was finally put in a *B*=9 T superconducting magnet, with the tube oriented in parallel with the magnetic field and left 2 h at *T*_room_ for the μLCEs to get aligned so that the nematic director pointed along the field, equivalently along the long axis of the sample. The superconducting magnet was used for efficiency reasons, since it provided for almost instant alignment, within several seconds, and for convenient and fast temperature control with its liquid nitrogen-operated continuous flow cryostat. The magnetic field-driven alignment has also been successfully tested in a *B*=1.2 T permanent magnet, however, with longer alignment times of several 10 min. Once aligned, the sample was left curing at *T*_0_≈50 °C<*T*_*λ*_ for about 12 h (see Supplementary Note 3 and Supplementary Fig. 4 for justification on the choice of *T*_0_). The resulting cured PDLCE specimens were cylindrical rods, 15 to 25 mm in length and 2.8 mm in diameter, with the look and feel of conventional PDMS (Fig. 1b). Disk-shaped samples were prepared in a similar way, using a sealed Teflon mould with a *ϕ*18, 2 mm high cylindrical cavity. Bilayer samples were made in two steps, by first setting the bottom layer and subsequently by fuse-depositing and setting the second layer on top of it. The orientation of thermomechanical anisotropy axis, equivalently of nematic director of oriented μLCEs, was controlled by individually orienting the mould with respect to external magnetic field for each layer.

### Thermomechanical characterization

*λ*(*T*) was measured in a home-built, step motor-driven and strain gauge-equipped thermomechanical analyser in a constant mechanical stress regime, *σ*≈0.2 kPa. A specimen was typically heated from *T*_room_ to 100 °C>*T*_*λ*_(*μLCE*-*A*) in the case of thermomechanically unimodal *PDLCE-A* and to 120°C>*T*_*λ*_(*μLCE*-*B*2) in the case of thermomechanically bimodal *PDLCE-B*. Measurements were then taken on cooling with a rate of about −10 °C per h down to *T*_room_. Young's modulus *E* of PDLCEs was determined from the tilt of the stress versus strain curve in the 0.2 to 5 kPa stress range. Partial moduli *E*_PDMS_ and *E*_LCE_ of the PDMS matrix and μLCEs were determined on bulk monodomain LCE stripes (typically 15 mm long, 5 mm wide, and 0.3 mm thick) and cured pure PDMS samples of the same geometry as PDLCEs (*ϕ*2.8 × 25 mm), respectively.

### Optimization of elastic properties

By systematically varying the base/hardener composition of the uncured PDMS resin, its relation with *E*_PDMS_ of the cured pure PDMS was determined and taken into account in fine-tuning the *E*_PDMS_ of the PDMS matrix component of PDLCEs. In particular, *E*_PDMS_ was found to decrease exponentially from 800 to 80 kPa on increasing the base share from 20:1 to 35:1. In order to maximize *λ*(*T*), all PDLCE composites, except for the ones used to investigate the dependence of *λ* and *E* on *y*=*E*_LCE_/*E*_PDMS_ (Fig. 8b,d), were prepared with 35:1 base/hardener composition of the PDMS matrix that exhibited a correspondingly low elastic modulus (*E*_PDMS_≈180 kPa). For compositions higher than 40:1, curing did not result in an elastically stable samples.

### Data availability

The data that support the findings of this study are available from the corresponding author upon request.

## Additional information

**How to cite this article:** Rešetič, A. *et al.* Polymer-dispersed liquid crystal elastomers. *Nat. Commun.* **7,** 13140 doi: 10.1038/ncomms13140 (2016).

## Supplementary Material

Supplementary Information Supplementary Figures 1-4, Supplementary Notes 1-3 and Supplementary References

Supplementary Movie 1 Animated simulation of fabrication and thermal actuation of PDLCEs with oriented L CE microparticles

Supplementary Movie 2 Thermal actuation of differently shape-change-programmed bilayer PDLCE-A specimens

Supplementary Movie 3 Beat-like thermal response of *PDLCE* - *B1/B2* specimen

## Figures and Tables

**Table 1:** List of bulk LCE and μLCE materials.

**Bulk LCE label**	**%wt** ***M4***	**%wt** ***M11***	**Crosslinking**	**External field**	**Bulk domain order**	**μLCE label**	**μLCE domains**
*LCE-A*	100	—	Two-step	Step 1: *σ*=0, *B*=0Step 2: *σ*≠0, *B*=0	Single crystal (fully ordered)	*μLCE-A*	Mono
*LCE-Ap*	100	—	Two-step	Step 1: *σ*=0, *B*=0Step 2: *σ*=0, *B*=0	Polydomain (disordered)	*μLCE-Ap*	Poly/mono
*LCE-B1*	60	40	Two-step	Step 1: *σ*=0, *B*=0Step 2: *σ*≠0, *B*=0	Single crystal (fully ordered)	*μLCE-B1*	Mono
*LCE-B2*	20	80	Two-step	Step 1: *σ*=0, *B*=0Step 2: *σ*≠0, *B*=0	Single crystal (fully ordered)	*μLCE-B2*	Mono
*LCE-C*	100	—	One-step	*σ*=0, *B*≠0	Partially ordered	*μLCE-C*	Mono
*LCE-Cp*	100	—	One-step	*σ*=0, *B*=0	Polydomain (disordered)	*μLCE-Cp*	Poly/mono

Shown are bulk LCE composition and fabrication parameters including the type of mesogens, crosslinking approach, external mechanical and magnetic field during crosslinking, and domain order of bulk materials, as well as domain character of their μLCE descendants.

**Table 2:** List of PDLCE composition and performance.

**PDLCE label**	**μLCE composition**	***B*** **during setting**	**μLCE order**	***λ*** **(*****T***_**0**_**)**
*PDLCE-A*	*μLCE-A*	1.2 T, 9 T	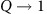	1.125±0.01
*PDLCE-Ap*	*μLCE-Ap*	9 T	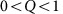	1.048±0.01
*PDLCE-A-iso*	*μLCE-A*	—	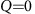	0.98±0.01
*PDLCE-Ap-iso*	*μLCE-Ap*	—	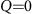	0.98±0.01
*PDLCE-B1*	*μLCE-B1*	9 T	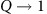	1.09±0.01
*PDLCE-B2*	*μLCE-B2*	9 T	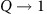	1.08±0.01
*PDLCE-B*	*μLCE-B1*, *μLCE-B2* (1:1)	9 T	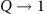	1.09±0.01
*PDLCE-C*	*μLCE-C*	9 T	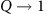	1.125±0.01
*PDLCE-Cp*	*μLCE-Cp*	9 T	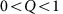	1.02±0.01

Shown are PDLCE composition and fabrication parameters including the type of microparticles, external magnetic field *B* during setting, estimated orientational order parameter 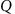, and thermomechanical performance *λ*(*T*_0_) (values taken from Figs 2). In particular, the morphable disks of Figs 4 and 5 are made of *PDLCE-A*, the bimodal specimen of Fig. 6 is made of *PDLCE-B*, whereas the layers of the beat-like response bilayer disk of Fig. 7 are made of *PDLCE-B1* and *PDLCE-B*2, respectively. The μLCE/PDMS mix resin (1:1 wt ratio) was set at *T*_0_=50 °C using 35:1 base/hardener composition of PDMS. The additional suffix ‘*-iso*' denotes isotropic PDLCEs obtained by setting the μLCE/PDMS resin in zero external magnetic field (*PDLCE-A-iso* and *PDLCE-Ap-iso*).
