# Supplementary material for: Polymer-dispersed liquid crystal elastomers
Source: Nat Commun. 2016 Oct 7;7:13140. doi: 10.1038/ncomms13140 (PMC5059788; doi:10.1038/ncomms13140)
Supplement: Supplementary Information — Supplementary Figures 1-4, Supplementary Notes 1-3 and Supplementary References [file ncomms13140-s1.pdf]

Supplementary Figures

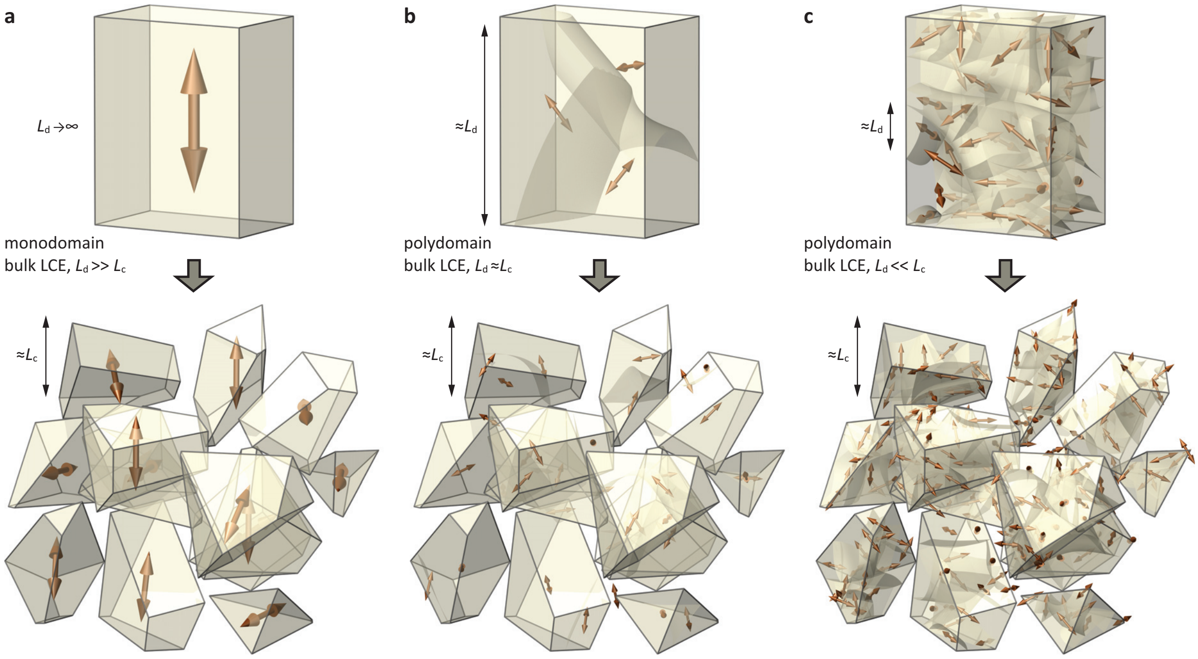

Supplementary Figure 1 | Preparation of LCE microparticles. Bulk LCE specimen are freeze-fractured into µLCE powder. Depending on the nematic domain order in the bulk, which can be either "single crystal"-like, e.g. monodomain (panel **a** top), polydomain with domain size L_d of the order of the µLCE size L_c (panel **b** top), or polydomain with domains much smaller than µLCE particles (panel **c** top), the resulting µLCEs are monodomain grains in the first case (panel **a** bottom), monodomain or low domain count grains in the second case (panel **b** bottom), and polydomain grains in the third case (panel **c** bottom).

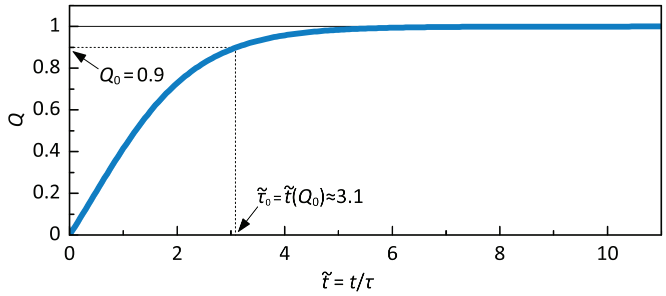

Supplementary Figure 2 | Time evolution of LCE microparticles' orientational order parameter. Theoretical $Q(\tilde{t} = t / \tau)$ profile is calculated from equation (13) of Supplementary Note 2.

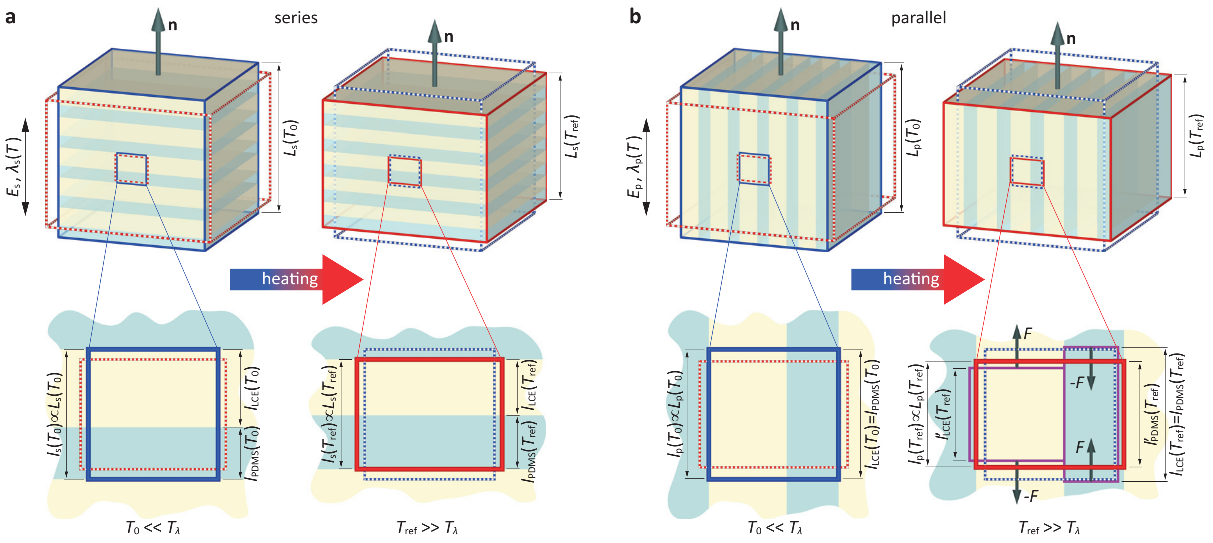

Supplementary Figure 3 | Sketch of the deformation of layer geometry on heating. a, “Series” scenario. **b**, “Parallel” scenario. The shape of the PDLCE specimen is shown below T_λ as a blue wireframe and above T_λ as a red wireframe. LCE and PDMS layers are shaded in light yellow and light green, respectively. The unconstrained shape (disregarded internal stress) is shown in magenta colour (panel **b** bottom right).

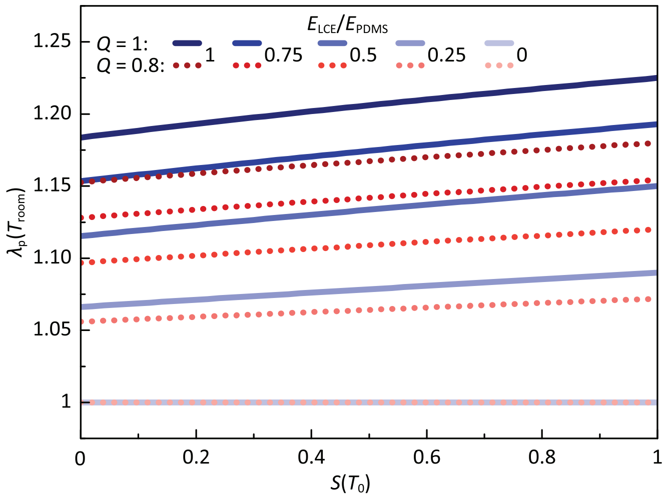

Supplementary Figure 4 | PDLCE performance. The effective thermomechanical response $\lambda_p(T_{room})$ depends on the nematic order parameter S of µLCE particles at the setting temperature T_0. Consecutive curves correspond to $y = E_{LCE} / E_{PDMS} = 0, 0.2,$ 0.4, 0.6, 0.8 and 1 (bottom to top), all calculated with $\gamma = 0.45$ and $\nu = 0.5$, with two different values of the µLCE orientational order parameter, $Q = 1$ for ideal alignment (blue solid lines) and $Q = 0.8$ for partially disordered alignment (red dashed line).

Supplementary Note 1 | Nematic domains in μLCEs

LCE microparticles, fabricated from fully ordered bulk LCEs, specifically from *LCE-A*, *LCE-B1*, and *LCE-B*2, are nematic monodomains (Supplementary Fig. 1), described as "mono" in Table 1. Crushing of polydomain bulk LCE materials (*LCE-Ap* and *LCE-Cp*), on the other hand, results in either polydomain or monodomain μLCEs (described as "poly/mono" in Table 1), depending on the actual size of microparticles (Supplementary Figs. 1b, c). In our case, typical sizes of μLCE and nematic domains both span the range of several microns to a few ten microns, so that μLCEs produced from polydomain bulk material consist of several misaligned nematic domains (Supplementary Fig. 1b) with small but non-zero residual effective nematic order S. In the partially ordered *LCE-C*, manufactured with magnetic field-assisted crosslinking, nematic domains are large enough for the freeze-fracturing to result in prevalently monodomain microsized LCE particles, with large residual S, irrespective of domain order of the bulk material. *LCE-C* thus serves equally well as the fully ordered bulk material like *LCE-A* in the production of monodomain μLCEs.

Supplementary Note 2 | Magnetic alignment

Orientational order of μLCEs. We shall assume that μLCE grains are ideal uniaxial nematic monodomains, so that their orientation is given by the angle $\vartheta\angle(\mathbf{n},\mathbf{Z})$ between the nematic director $\mathbf{n}$ and the $\mathbf{Z}$ axis of the laboratory frame. The orientational distribution $P(\cos\vartheta)$ of μLCE grains, which is isotropic in the prepolymer mixture, $P_0(\cos\vartheta)=1,$ becomes increasingly anisotropic when LCE microparticles are aligned in the external magnetic field,

$$P_t(\cos\vartheta)=\int_0^1 \delta\left[\cos\vartheta-\cos\vartheta(t,\,\cos\vartheta_0)\right]\,d\cos\vartheta_0\,. \tag{1}$$

Here t measures the time since the start of aligning and δ denotes the Dirac's delta function. Relation $\cos\vartheta(t,\cos\vartheta_0),$ which is obtained by solving the equation of motion, measures the alignment of a given LCE microparticle with its nematic director initially oriented at $\vartheta_0\angle(\mathbf{n},\mathbf{B}\,||\,\mathbf{Z})$ at time $t=0.$ We have taken into account that, during the aligning, $P_t(\cos\vartheta)$ remains (i) cylindrically symmetric, i.e. independent of azimuthal angle $\varphi,$ about $\mathbf{B}$ and (ii) invariant to the transformation $\vartheta\rightarrow\pi-\vartheta,$ allowing for restriction of tilt angle definition range to $\cos\vartheta\in[0,1].$ The orientational order is commonly quantified by the orientational order parameter $Q=\overline{(3\cos^2\vartheta-1)/2},$ with the average performed over $P(\cos\vartheta)$ (Supplementary Ref. 1). In our specific case, taking into account equation (1), we obtain

$$Q(t)=\frac{1}{2}\int_0^1\left[3\cos^2\vartheta(t,\,\cos\vartheta_0)-1\right]\,d\cos\vartheta_0\,. \tag{2}$$

PDLCEs can thus be simply characterized in terms of $Q,$ which quantifies the orientational order of μLCEs. For PDLCE composites, cured in the external field $\mathbf{B},$ full alignment $(Q\rightarrow1)$ or partial alignment $(0<Q<1)$ is achieved in the respective cases of monodomain or polydomain μLCEs. On the other hand, during zero field curing, LCE microparticles remain isotropically distributed $(Q=0),$ therefore we label this state as "*iso*" (Table 2). Consistently, PDLCE specimens with $Q>0$ exhibit macroscopically observable thermomechanical response, whereas PDLCE specimens with $Q=0$ are thermomechanically inert (Fig. 2).

Equation of reorientational motion. LCE microparticles are treated as diamagnetically anisotropic, nematic monodomain ellipsoidal objects. Their alignment is driven by the magnetic torque

$$\Gamma_B=-\frac{1}{\mu_0}VS\Delta\chi B^2\sin\vartheta\,\cos\vartheta \tag{3}$$

and viscous torque

$$\Gamma_\eta=-\frac{8\pi\eta r^3}{F(r/R)}\frac{d\vartheta}{dt}. \tag{4}$$

Here μ_0 denotes the magnetic permeability of the vacuum, V the volume of the microparticle, S the nematic order parameter, $\Delta\chi$ the anisotropy of diamagnetic susceptibility, $\vartheta\angle(\mathbf{n},\mathbf{B})$ the angle between the nematic director $\mathbf{n}$ and magnetic field $\mathbf{B}$, and η the viscosity of the uncured matrix. r and R are the radii of the short and long axis of the ellipsoid, respectively. The geometrical factor $F(r/R)$ equals 1 for a sphere $(r/R=1)$ and exhibits only a modest decrease for moderately deformed sphere, e.g.

$F(0.5) \approx 0.9$, $F(2) \approx 0.7$ (Supplementary Ref. 2). Since our particular LCE microparticles resemble spheres rather than rods or discs, we accordingly approximate $r \approx R \Rightarrow F \approx 1$. The equation of motion then reads

$$I \frac{d^2 \vartheta}{dt^2} = \Gamma_B + \Gamma_\eta . \tag{5}$$

$I = 2\rho V R^2 / 5$ is the sphere's moment of inertia and ρ the volumetric mass density of μLCEs. By defining two characteristic times,

$$\tau = \frac{6\eta\mu_0}{S\Delta\chi B^2} \tag{6}$$

and

$$\tau_R = \frac{\rho R^2}{15\eta}, \tag{7}$$

one can rewrite equation (5) into

$$\tau\tau_R \frac{d^2\vartheta}{dt^2} = -\sin\vartheta \cos\vartheta - \tau\frac{d\vartheta}{dt}. \tag{8}$$

The impact of particle size R on the motion is best estimated by introducing dimensionless time $\tilde{t} = t / \tau$ and dimensionless acceleration coefficient $\alpha = \tau_R / \tau = R^2 / R_0^2$, the magnitude of the latter determined by the characteristic radius

$$R_0 = \frac{3\eta}{B}\sqrt{\frac{10\mu_0}{\rho S \Delta\chi}}. \tag{9}$$

Using such a notation, equation (8) becomes

$$\alpha \frac{d^2\vartheta}{d\tilde{t}^2} = -\sin\vartheta \cos\vartheta - \frac{d\vartheta}{d\tilde{t}}, \tag{10}$$

implying that for $\alpha \ll 1$, equivalently $R_0 \gg R$, the acceleration term on the left can be disregarded. For our specific choice of materials, shown in Fig. 1d, $\eta \approx 3.5\ \mathrm{Pa\ s}$ for PDMS, $\rho \approx 10^3\ \mathrm{kg\ m^{-3}}$, $S(T_{\mathrm{room}}) \approx 0.65$ (Supplementary Ref. 3), and $\Delta\chi \approx 10^{-7}$ (Supplementary Ref. 4) for *M4*-based *μLCE-A*, yielding $R_0 \gtrsim 0.5\ \mathrm{m}$ in the external magnetic field $B \lesssim 9\ \mathrm{T}$. α is thus vanishingly small for μLCE particles which are sized below 100 μm. Consequently, their reorientational dynamics obeys the relation[5]

$$\frac{d\vartheta}{d\tilde{t}} = -\sin\vartheta \cos\vartheta. \tag{11}$$

With initial conditions $d\vartheta / d\tilde{t}(\tilde{t} = 0) = 0$ (microparticles are still when B is turned on), $\vartheta(\tilde{t} = 0) = \vartheta_0$ (a given microparticle is initially oriented at an arbitrary angle ϑ_0) and assuming $\Delta\chi > 0 \Rightarrow \tilde{t}(t > 0) > 0$, an analytical solution

$$\cos\vartheta\left(\tilde{t},\,\cos\vartheta_0\right)=\frac{1}{\sqrt{1+\dfrac{1-\cos^2\vartheta_0}{\cos^2\vartheta_0}\mathrm{e}^{-2\tilde{t}}}} \tag{12}$$

is obtained that results in (see relation (2))

$$Q\left(\tilde{t}\right)=\frac{\sqrt{\mathrm{e}^{2\tilde{t}}-1}\left(1+2\mathrm{e}^{2\tilde{t}}\right)-3\mathrm{e}^{2\tilde{t}}\arctan\sqrt{\mathrm{e}^{2\tilde{t}}-1}}{2\left(\mathrm{e}^{2\tilde{t}}-1\right)^{3/2}}. \tag{13}$$

Alignment timescale. The resulting $Q(\tilde{t})$ increases monotonously in time, with limiting values $Q(\tilde{t}=0)=0$ for initial isotropic distribution at $t=0$ and $Q(\tilde{t}\to\infty)=1$ for fully aligned LCE microparticles at $t\gg\tau$ (Supplementary Fig. 2). By choosing $Q=0.9$ as a criterion for satisfactory alignment, the respective alignment time is estimated as $\tau_0=t(Q=0.9)\approx 3.1\tau$. Taking into account relation (6), we finally derive

$$\tau_0=\frac{\beta}{SB^2},\ \ \beta\approx\frac{18\eta\mu_0}{\Delta\chi}. \tag{14}$$

With the above listed values of η, $\Delta\chi$, and S we obtain $\beta/S\lesssim 1.3\times10^3\,\mathrm{s\,T^2}$, specifically $\tau_0(B=1\,\mathrm{T})\approx 22$ min and $\tau_0(B=9\,\mathrm{T})\approx 17$ s.

Supplementary Note 3 | Modelling of thermomechanical response

Serial and parallel models of a binary composite. We shall calculate the effective thermomechanical response $\lambda(T)$ of a PDLCE specimen by considering a simple scenario of a binary composite with alternating layers of an isotropic elastomer (PDMS in our specific case) and thermomechanically active LCE, stacked in series (index s) or in parallel (index p) with respect to the orientation of nematic director $\mathbf{n}$ of the LCE component (Supplementary Fig. 3). It is straightforward to show that the respective elastic moduli along the director $\mathbf{n}$ can be expressed as[6]

$$E_{\mathrm{s}} = E_{\mathrm{LCE}} \Big/ \left[\nu + (1-\nu)y\right] \tag{15a}$$

and

$$E_{\mathrm{p}} = E_{\mathrm{LCE}} \left[\nu + (1-\nu)y\right], \tag{15b}$$

with ν denoting the volume fraction of μLCE inclusions and $y = E_{\mathrm{LCE}} / E_{\mathrm{PDMS}}$ the relative elasticity of the LCE material with respect to the PDMS matrix. Let us now calculate the corresponding thermomechanical responses $\lambda_{\mathrm{s}}(T)$ and $\lambda_{\mathrm{p}}(T)$ with the help of Supplementary Fig. 3, which depicts the changes in layer geometry with temperature for the "series" and "parallel" models. Taking into account that, at the resin setting temperature T_0, the internal stress between the layers vanishes, we select at T_0 a square-shaped region containing an LCE and a PDMS layer with respective cross-section areas A_{LCE} and A_{PDMS}. This region deforms into a rectangle at a general temperature T (solid line frames in Supplementary Fig. 3). We disregard the changes of layer geometry in the plane perpendicular to $\mathbf{n}$ (A_{LCE} and A_{PDMS} independent of T). If the two layers were unconstrained, their lengths along $\mathbf{n}$ would exhibit respective thermal profiles $L_{\mathrm{LCE}}(T)$ and $L_{\mathrm{PDMS}}(T)$. The effective length of the composite layer can then be written as

$$L_{\mathrm{s}}(T) = L_{\mathrm{LCE}}(T) + L_{\mathrm{PDMS}}(T) \tag{16}$$

for the "series" model and

$$L_{\mathrm{p}}(T) = L_{\mathrm{LCE}}(T) = L_{\mathrm{PDMS}}(T), \tag{17a}$$

$$L_{\mathrm{LCE}}(T) = L'_{\mathrm{LCE}}(T)\left[1 + \frac{F(T)}{E_{\mathrm{LCE}} A_{\mathrm{LCE}}}\right], \tag{17b}$$

$$L_{\mathrm{PDMS}}(T) = L'_{\mathrm{PDMS}}(T)\left[1 - \frac{F(T)}{E_{\mathrm{PDMS}} A_{\mathrm{PDMS}}}\right] \tag{17c}$$

for the "parallel" model where the length mismatch between $L'_{\mathrm{LCE}}(T)$ and $L'_{\mathrm{PDMS}}(T)$ is compensated by respective internal stresses $F(T)/A_{\mathrm{LCE}}$ and $-F(T)/A_{\mathrm{PDMS}}$. These vanish at T_0 since $F(T_0) = 0$. This also implies

$$L'_{\mathrm{LCE}}(T_0) = L'_{\mathrm{PDMS}}(T_0). \tag{17d}$$

We shall define the thermomechanical response with respect to the reference temperature T_{ref}. This is conventionally chosen to be above the temperature T_λ characterizing the anomaly in the $L_{\mathrm{LCE}}(T)$ profile due to the onset of nematic order $(S \neq 0)$ in μLCE particles. The effective thermomechanical response of the PDLCE composite it thus introduced as

$$\lambda(T) = \frac{L(T)}{L(T_{\text{ref}})} \tag{18a}$$

and the individual responses of the two components as

$$\lambda_{\text{LCE}}(T) = \frac{L_{\text{LCE}}(T)}{L_{\text{LCE}}(T_{\text{ref}})} \tag{18b}$$

and

$$\lambda_{\text{PDMS}}(T) = \frac{L_{\text{PDMS}}(T)}{L_{\text{PDMS}}(T_{\text{ref}})}. \tag{18c}$$

The μLCE volume fraction

$$v = \frac{A_{\text{LCE}} L_{\text{LCE}}(T_0)}{A_{\text{LCE}} L_{\text{LCE}}(T_0) + A_{\text{PDMS}} L_{\text{PDMS}}(T_0)} \tag{19}$$

can be expressed as

$$v_{\text{s}} = \frac{L_{\text{LCE}}(T_0)}{L_{\text{LCE}}(T_0) + L_{\text{PDMS}}(T_0)} \tag{20a}$$

in the "series" scenario, by assuming T-independent $A_{\text{LCE}} = A_{\text{PDMS}}$. In the "parallel" scenario, on the other hand, relation (17a) results into

$$v_{\text{p}} = \frac{A_{\text{LCE}}}{A_{LCE} + A_{\text{PDMS}}}. \tag{20b}$$

We now insert L_{s} of relation (16) in place of L in relation (18a) and take into account relations (18b) and (18c), with $L_{\text{LCE}}(T_0) / L_{\text{PDMS}}(T_0) = v / (1 - v)$ implied by (20a), to obtain the "series" thermomechanical response $\lambda_{\text{s}}(T)$. Similarly, by using L_{p} of relation (17a) in place of L with eliminated $F(T)$ and $A_{\text{LCE}} / A_{\text{PDMS}} = v / (1 - v)$ derived from (20b), we obtain the "parallel" analogue $\lambda_{\text{p}}(T)$. The results are

$$\lambda_{\text{s}}(T) = \frac{v\lambda_{\text{LCE}}(T)\lambda_{\text{PDMS}}(T_0) + (1-v)\lambda_{\text{LCE}}(T_0)\lambda_{\text{PDMS}}(T)}{v\lambda_{\text{PDMS}}(T_0) + (1-v)\lambda_{\text{LCE}}(T_0)} \tag{21a}$$

and

$$\lambda_{\text{p}}(T) = \frac{(1-v)\lambda_{\text{PDMS}}(T_0) + yv\lambda_{\text{LCE}}(T_0)}{(1-v)\lambda_{\text{PDMS}}(T_0) / \lambda_{\text{PDMS}}(T) + yv\lambda_{\text{LCE}}(T_0) / \lambda_{\text{LCE}}(T)}. \tag{21b}$$

We note that the above two expressions can be applied to any binary composite of elastic components, since no specific temperature profiles for $\lambda_{\text{LCE}}(T)$ and $\lambda_{\text{PDMS}}(T)$ have been assumed so far. The combined response is a combination of the two individual responses weighted by v and y. It also depends on the setting temperature T_0. Relations (15a), (15b), (21a) and (21b) at $T = T_{\text{room}}$ were used to generate the theoretical curves of Figs. 8a-d.

Impact of PDLCE setting temperature T_0. Let us now evaluate the role of the setting temperature T_0, particularly in view of maximizing thermomechanical response of the PDLCE composite. In the following we only consider the "parallel" scenario, which is superior to the "serial" scenario in reproducing our experimental data. The explicit temperature dependences of $\lambda_{\mathrm{LCE}}(T)$ and $\lambda_{\mathrm{PDMS}}(T)$ can be written as

$$\lambda_{\mathrm{LCE}}(T)=\frac{L_{\mathrm{LCE}}(T_{\mathrm{ref}})+\Delta L_{\mathrm{LCE}}^{(S)}(T)+\Delta L_{\mathrm{LCE}}^{(\alpha)}(T)}{L_{\mathrm{LCE}}(T_{\mathrm{ref}})}=1+\gamma QS(T)+\alpha_{\mathrm{LCE}}(T-T_{\mathrm{ref}}) \tag{22a}$$

and

$$\lambda_{\mathrm{PDMS}}(T)=\frac{L_{\mathrm{PDMS}}(T_{\mathrm{ref}})+\Delta L_{\mathrm{PDMS}}^{(\alpha)}(T)}{L_{\mathrm{PDMS}}(T_{\mathrm{ref}})}=1+\alpha_{\mathrm{PDMS}}(T-T_{\mathrm{ref}}), \tag{22b}$$

respectively. The $\gamma S(T)$ term arises from the coupling between the orientational order of nematogenic molecules, characterized by $S(T)$, and the polymer backbone[7]. This coupling is absent in the elastically isotropic PDMS. The additional multiplicator Q is used speculatively to encompass the scenario of non-ideally aligned µLCE particles ($Q<1$, see Supplementary Note 2). We observe that $\lambda_{\mathrm{LCE}}(T)$ given by expression (22a) is the µLCE material property, whereas Q is the collective property of the ensemble of µLCE particles embedded in the PDMS matrix. Nevertheless, the effective behaviour of PDLCE is properly reproduced, since with $Q=0$, i.e. with isotropically distributed µLCE, the Q-weighted $\lambda_{\mathrm{LCE}}(T)$ becomes insensitive to the onset of the nematic order $S(T)$. The $\alpha(T-T_{\mathrm{ref}})$ term describes conventional linear thermal expansion. Specifically, $\alpha_{\mathrm{PDMS}}\approx\alpha_{\mathrm{LCE}}\approx 7\times10^{-4}\,\mathrm{K}^{-1}$, as calculated from thermomechanical response curves of Fig. 2. γ can be determined experimentally from the relation $\gamma\equiv\lambda_{\mathrm{LCE}}(T(S\to1))-1-\alpha_{\mathrm{LCE}}(T(S\to1)-T_{\mathrm{ref}})$. For *µLCE-A*, $T(S\to1)\approx T_{\mathrm{room}}$ and $\lambda_{\mathrm{LCE}}(T_{\mathrm{room}})\approx1.4$, yielding $\gamma\approx0.45$. We can disregard the linear temperature expansion, i.e. $\alpha_{\mathrm{LCE}}=\alpha_{\mathrm{PDMS}}=0$, without sacrificing any important aspects of the dependence of $\lambda_{\mathrm{p}}(T)$ on T_0. Equation (21b) then rewrites to

$$\lambda_{\mathrm{p}}(T)=\frac{1-v+yv\left[1+\gamma QS(T_0)\right]}{1-v+yv\left[1+\gamma QS(T_0)\right]/\left[1+\gamma QS(T)\right]}. \tag{23}$$

$\lambda_{\mathrm{p}}(T)$ increases with increasing nematic order parameter $S(T)$ of the µLCE component, i.e. on lowering the temperature, and is maximized at $S=1$. Supplementary Fig. 4 demonstrates that the maximized value $\lambda_{\mathrm{p}}(S=1)$, which can be approximated by $\lambda_{\mathrm{p}}(T_{\mathrm{room}})$ for all LCE materials of Table 1, only slightly depends on the order parameter $S(T_0)$ at the resin setting temperature T_0. The setting could thus be in principle performed either in the nematic phase or in the isotropic phase. However, the magnetic alignment is ineffective in the isotropic phase since there the characteristic alignment time diverges, $\tau_0(S\to0)\to\infty$ (relation (14) of Supplementary Note 2). In conclusion, the alignment is best preserved in the final composite when the curing temperature T_0 is kept constant and well below the nominal temperature T_λ of the thermomechanical anomaly of µLCEs during thermal setting (this is why we chose $T_0=50°\mathrm{C}$).

Supplementary References

1. Lebar, A., Kutnjak, Z., Žumer, S., Finkelmann, H., Sánchez-Ferrer A. & Zalar, B. Evidence of supercritical behaviour in liquid single crystal elastomers. *Phys. Rev. Lett.* **94**, 197801 (2005).
2. Doi, M. & Edwards, S. F. *Theory of Polymer Dynamics* (Clarendon Press, Oxford, 1986).
3. Milavec, J., Domenici, V., Zupančič, B., Rešetič, A., Bubnov, A. & Zalar, B. Deuteron NMR resolved mesogen vs. crosslinker molecular order and reorientational exchange in liquid single crystal elastomers. *Phys. Chem. Chem. Phys.* **18**, 4071-4077 (2016).
4. Schad, Hp., Baur, G. & Meier, G. Investigation of the dielectric constants and the diamagnetic anisotropies of cyanobiphenyls (CB), cyanophenylcyclohexanes (PCH), and cyanocyclohexylcyclohexanes (CCH) in the nematic phase. *J. Chem. Phys.* **71**, 3147-3181 (1979).
5. Kimura, T., Yamato, M., Koshimizu, W., Koike, M. & Kawai, T. Magnetic orientation of polymer fibers in suspension. *Langmuir* **16**, 858-861 (2000).
6. Daniel, I. & Ishai, O. *Engineering Mechanics of Composite Materials* (Oxford Univ. Press, 2007).
7. Warner, M. & Terentjev, E. M. *Liquid Crystal Elastomers* (Oxford Univ. Press, 2007).
